# Project Report on Telemedicine: What We Learned about the Administration and Development of a Binational Digital Infrastructure Project

**DOI:** 10.3390/healthcare9040400

**Published:** 2021-04-01

**Authors:** Norbert Hosten, Britta Rosenberg, Andrzej Kram

**Affiliations:** 1 Department of Radiology, Universitätsmedizin Greifswald, Fleischmannstraße 8, 17475 Greifswald, Germany; norbert.hosten@med.uni-greifswald.de; 2 Department of Pathology, Westpomeranian Oncology Center, Strzałowska 22, 71-730 Szczecin, Poland; akram@onkologia.szczecin.pl

**Keywords:** telemedicine, telediagnostic, project management, funding, IT infrastructure, cross-border multiprofessional team, European Union, Interreg

## Abstract

This article describes the development of a German–Polish cross-border telemedicine project. Funded by the European Union Interreg Program, a cooperation between several German and Polish hospitals was developed over the course of 16 years, starting in 2002. Subprojects, governance and outcomes are described, and facilitators and barriers are identified. These points are reviewed with regard to their influence on medical, technical, administrative and medico-legal realisation.

## 1. Introduction

This report explores the lessons learnt from a German–Polish telemedicine network funded by the European Union (EU) in Pomerania. 

Pomerania is a historic region on the southern shore of the Baltic sea, with a western part located in Germany and an eastern part located in Poland. The area of historic Pomerania was used to create a co-operation structure between Germany and Poland in 1995 (“Euroregion Pomerania”). Ten German cities and districts and 98 Polish municipalities are members of the Euroregion. The purpose of all Euroregions is the promotion of common interests. Euroregions are eligible for funding in the Interreg programs of the European Union. Interreg I started in 1989. The present phase of the program, Interreg V, funded projects until 2020. Interreg was created to promote cross-border cooperation in the EU, thus diminishing the influence of national borders.

The council of the Pomeranian Euroregion is located in (Polish) Szczecin, the historic capital of ancient Pomerania. The council is constituted in equal parts by German and Polish members. Polish members of the Euroregion are members of a Polish association, while German members belong to a “Kommunalgemeinschaft”—an association according to German law.

Eligible projects may apply for funding from current Interreg programs. Only groups that are constituted by both German and Polish members are suitable for application. A lead partner may be either from Germany or Poland. The agency in the lead partner’s country of origin will then process the request, aiming to achieve harmonisation between the Polish and the German side.

A complete review of Interreg is beyond the scope of this paper. Perkmann [1] gives an overview of the concept and existing cross-border regions, following Schmitt-Egner’s [2] definition of ‘cross-border cooperation’ as ‘cross-border interaction between neighbouring regions for the preservation, governance and development of their common living space, without the involvement of their central authorities” (Perkmann’s translation).

**Figure 1:**
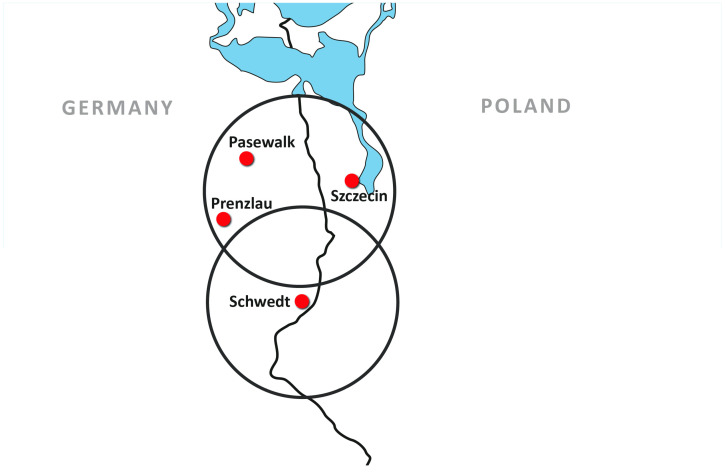
Influence of the boundary between Germany and Poland on the catchment areas of hospitals in the cities of Szczecin, Pasewalk, Prenzlau and Schwedt. It is apparent that the hospitals close to the border have small catchment areas and that there are areas without easily accessible hospitals.

The rationale for implementing telemedicine in the Euroregion of Pomerania was twofold: (1) The regions on both sides of the German/Polish border are very thinly populated. The German federal state of “Vorpommern” (Western Pomerania) has a population density of 69 inhabitants/square kilometre, while the Polish voivodeship’s (province) “województwo zachodniopomorskie”(Voivodeship Western Pomerania) has 75 inhabitants/square kilometre (mean population density: Germany, 137 inhabitants/square kilometre; Poland, 132 inhabitants/square kilometre). (2) The border in the Pomerania region between Poland and Germany leaves several German and Polish hospitals with small catchment areas (Figure 1). Telemedicine is an accepted means of delivering medical services to people in such areas by enlarging catchment areas [3,4].

Accordingly, people living in these regions may have reduced access to hospitals. To improve access to specialised medicine, a joint telemedicine project was initiated starting in 2002. With Interreg IVA funding, the most recent phase of this project was initiated with 11 German and 11 Polish hospitals. Specialities taking part were radiology, pathology, ophthalmology, urology and otorhinolaryngology (ear–nose–throat medicine, ENT) as well as radiation therapy, oncology and thoracic surgery in tumour boards. On the German side, an extensive videoconferencing network was laid out, which allowed for the simultaneous transport of x-ray studies, pathology slides, endoscopy images and documents.

As medicine is generally organised nationally, telemedicine tends also to be national. The project described here originated in radiology and pathology, with both of them dealing with physician-to-physician interaction; another focus was videoconferencing. On the German side, an expensive infrastructure had to be bought and installed to support these applications. Management, facilitators and barriers for these parts of the project are described below. We report here on the lessons learned from the implementation of this Interreg IV project.

## 2. Materials and Methods

### 2.1. Previous Phases of the Project

The project described here was implemented over the course of four funding periods since 2002. The first author (a university radiologist, chairman of the project’s board) has been involved from the beginning; the other two authors (in-house counsel and pathologist) have been involved since 2010. Documentation from the different project phases was used for analysis. Because public funds were used, the written documentation was comprehensive. The work presented here aims to describe facilitators and barriers and thus to provide guidance for colleagues wishing to implement similar international projects. The paper focuses on the last phase of the project which started in 2010. There were three previous project phases, each with six-digit funding (all figures in Euros):

1. A digitisation project focusing on radiology and pathology in Pomerania (“Pommern”, Germany) and Poznań.
2. A regional expansion of the digitisation project to North Brandenburg and Poland.
3. Another digitisation phase between Pomerania, northern Brandenburg and Poznań.
4. Beginning in 2010, the current project between Pomerania, northern Brandenburg and the voivodeship of Western Pomerania and Poznań (a fifth phase of the project with cross-border patient treatment has been approved; it is mentioned here as it aims at the cross-border treatment of patients (children with neuroblastoma). Grounded in the first three phases, the project was required to develop a structure that allowed for the implementation of a telemedicine network in a larger state.

**Figure 2:**
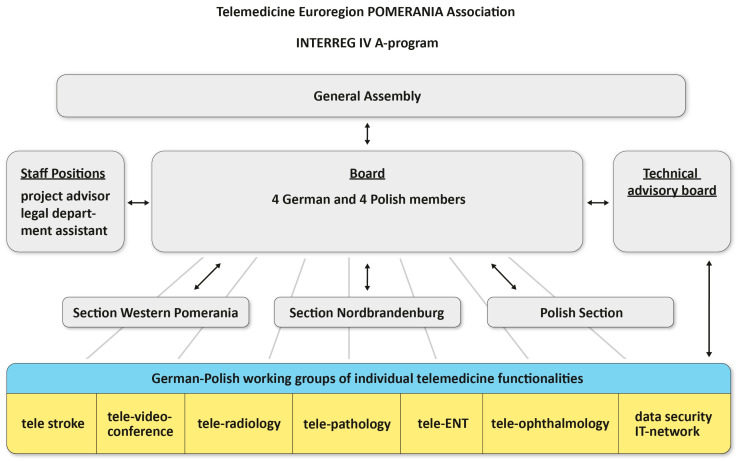
Organigram of the association “Telemedicine Euroregion Pomerania”. The German project participants organised themselves in an association under German law; three Polish members were co-opted. Their assembly (one representative with voting rights from each of the participating hospitals) approved key decisions and the budget once a year. The assembly elected the association’s board. Additional Polish members took part in German–Polish working groups. The German side was the lead partner of the project. It organised the settlement of the funds. The board of directors had several employees for legal, financial and secretarial tasks. A technical advisory board staffed with independent technical experts met twice a year for three years to review the investments.

The following documents from project phases 1 to 4 were available for analysis:

- From business plans, grant approvals and project applications, we extracted and evaluated objectives and the amount of funding (a low eight-digit amount of funding in the phase described here).
- Accountability reports were available for the project phases between 2002 and 2010. They were evaluated for an overview of newly installed devices.
- Presentation slides.
- For the fourth phase of the project, the following documents were additionally available:
  - The association’s statutes, with descriptions of the organisational structure (Figure 2); protocols of the yearly assembly between 2010 and 2020.
  - The protocols of the meetings of the Board of Directors between 2010 and 2016.
  - The business plan for the association from 2010.
  - The protocols of the technical advisory board’s meetings, which had to approve purchases.
  - Medico-legal and medico-economic analyses as published by the project in specialist journals on the projects: the tele-tumour conference, teleradiology, telepathology, tele-ENT (overview lecture, published), Tele-Glaucoma (technical description) and Tele-Stroke.
  - Final reports to the sponsor.

### 2.2. Problems Identified in Ihe Scientific Literature on Telemedicine That Were Evaluated

The following text focuses on lessons learned from the Pomerania telemedicine cross-border project. Facilitators and barriers encountered during the implementation of telemedicine in rural regions have been covered in the literature [3,4,5,6,7]. There is, however, hardly any mention of management and organisation matters in such projects. The following barriers are named, among others (they also turned out to be central to our project): high capital expenditure overheads, a lack of motivation and financial benefits for application developers and telehealth service providers, a lack of a strategy to transform telehealth trials into sustainable real-world services, insufficient financial support through government reimbursement (e.g., to buy telehealth equipment) and unmet requirements to train people to deal with cultural differences.

### 2.3. Participating Hospitals Whose Projects Were Analysed

**Figure 3:**
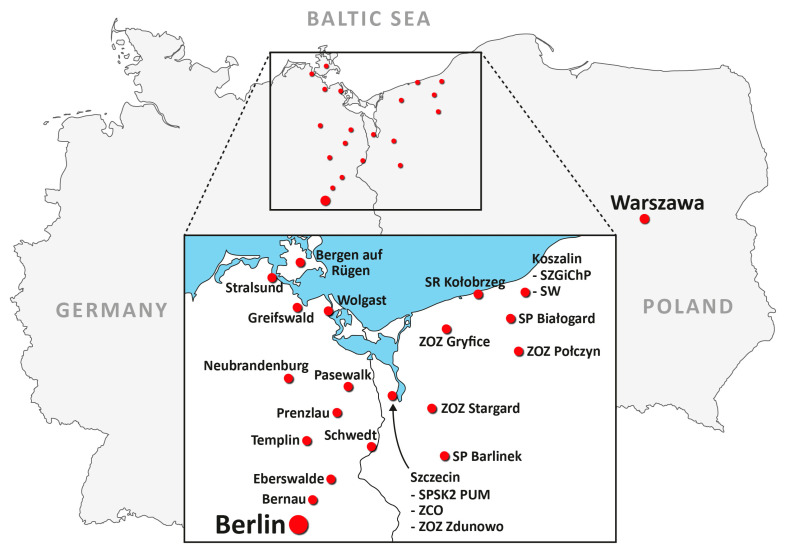
Geographical distribution of hospitals on both sides of the German–Polish border. Berlin, Warsaw and the Baltic Sea are also indicated for the better visualisation of the project.

The participating hospitals on the Polish side were SPSK2 PUM Szczecin, ZCO Szczecin, ZOZ Zdunowo Szczecin, SP Barlinek, SR Kołobrzeg, SZGiChP Koszalin, SW Koszalin, ZOZ Stargard, ZOZ Gryfice, ZOZ Połczyn, and SP Białogard; on the German side, the hospitals were Sana Bergen/Rügen, Asklepios Stralsund, Universitätsmedizin Greifswald, Krankenhaus Wolgast, Asklepios Pasewalk, Dietrich-Bonhoeffer-Klinikum Neubrandenburg, GLG Eberswalde, GLG Prenzlau, Asklepios Schwedt, Sana Templin, and Herzzentrum Bernau (Figure 3).

## 3. Results

### 3.1. Results Regarding Organisation of the Project

To develop a structure for a telemedicine network, the Telemedicine Association in the Euroregion of Pomerania was founded in 2008. It was funded in the Interreg program from 2010 onwards. According to German law, an association is not primarily dedicated to generating profits; it receives public funding more easily than a limited company. The association was registered with statutes, and hospitals were invited to a constituent meeting. In the next step, a business plan was drawn up. This budgeted the establishment of an office (see Figure 2) and the financing of staff beyond funding. The association was provided with a low five-digit capital. An IT consultancy (DFC, Munich-Germany) was commissioned to develop a concept for the German side of the funding area. Under the umbrella term “telemedicine”, the concept planned the modalities listed in Table 1. They were underlaid with digitised medical devices and equipment for storage, network connection, etc. After several rounds of negotiations with the relevant Ministry of Economics, the concept was accepted and the project was funded.

The EU’s outcome parameters differ from the clinical/medical parameters discussed below. Parameters and eligibility requirements for the Interreg program are summarised in manuals. In addition to the basic requirement (beneficiaries from at least two participating countries, at least one of which is a member state of the EU), the following methods of cooperation are also required:

1. Joint conceptualisation, which may for example be achieved by holding regular project development meetings, establishing institutionalised long-term contacts, joint project preparation and/or scheduling.
2. Joint implementation, which may for example be achieved by joint management or partial responsibilities for each of the project partners.
3. Joint staffing.
4. Joint funding.

It is important that cross-border cooperation in the specific program context does not have to consist of cross-border patient treatment—a criterion often expected, in particular by the press. The relative freedom in designing the project with the total amount capped at a low eight-digit amount led to the establishment of a technical advisory board on the German side. This independent, national committee of experts had to approve all investments in advance. The State Court of Auditors then reviewed and accepted the entire project financing.

The commitment to fund the office and staff beyond the funding phase was the decisive factor in the project’s eligibility. An annual budget was adopted at each of the annual general meetings.

The typical EU outcome parameters are the numbers of persons reached and the amount and quality of the publicity. The facilitator for fulfilling expectations by the EU was the German–Polish structure. This was a definitive advantage for the public perception of the project in Germany. The project was, e.g., visited by the then Federal President Gauck. This generated much publicity. For the organizing IT company, the project was important beyond the level of income due to national visibility. As with the medical community, the IT industry has its own communication channels; the project received also received good press in this context. At the major German trade fairs (Medica, Düsseldorf; conhIT Berlin) there were opportunities to present the project that are not readily available to single-site telemedicine projects. A barrier was the perception of the association as a parallel structure by hospital administrations; the expansion of competencies by physicians was suspected.

### 3.2. Results by Telemedical Specialty

An overview of subprojects, outcome indicators, outcomes and facilitators/barriers is given in Table 1.

#### 3.2.1. Videoconferencing Network

Telemedical interactions between people benefit from an image transmission that provides facial expressions as well as the other person’s spoken language. Naturally, this also applies to interactions between physicians. Common videoconferencing systems, which in the meantime have become widely available to all, can be used for these image transmissions. Data safety has to be ensured when using these devices. Various studies have shown that patients are willing to communicate with their doctors via videoconferencing [8,9]. The prerequisite, however, is that the advantages outweigh the disadvantages. Perhaps the most relevant advantage in particular is saving time due to the elimination of physical transportation from the communication process. In the planning process of the project, we assumed that this would also be the point of view of doctors communicating with colleagues. 

**Figure 4:**
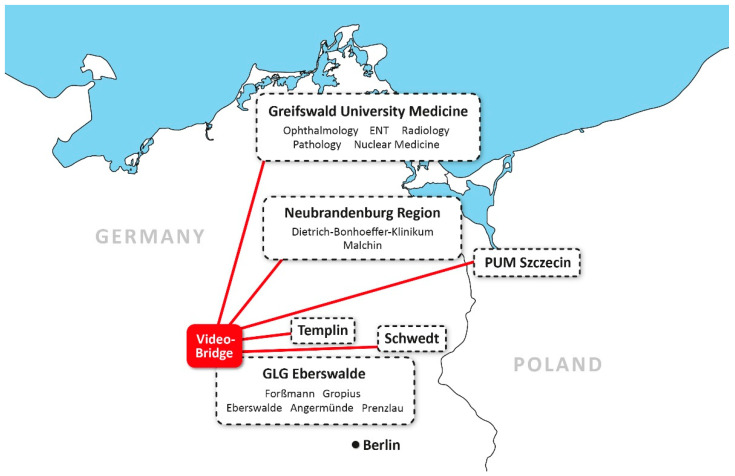
A videoconferencing network was the backbone of the telemedicine project. On the German side, there was a northern (Greifswald), a central (Neubrandenburg) and a southern rail (Eberswalde) with videoconferencing links; in Poland, only Szczecin took part. A “bridge”, actually a switch allowing multi-point videoconferencing to be initiated, was located in the south rail. This limited use of the network required three bridges to be installed. The system also allowed for the simultaneous viewing of medical images (x-ray, real-time endoscopy and pathology slides) and various documents on additional monitors. Please note that not all hospitals that participated in the program also participated in the videoconferencing network, explaining the difference in numbers.

Within the project, a videoconferencing system was installed that could connect participants via a “video-bridge” (Figure 4). This system that consisted of 15 sites (14 German and 1 Polish; not all hospitals participated, but some hospitals had more than one site) was the backbone of the entire project. The following situations were covered:

- A tele-tumour conference connected several hospitals and allowed tumour conferences to be held with several specialists.
- Various tumour conferences within a hospital allowed the involvement of specialists (e.g., pathologists) who had only a few points to make for one or two minutes per hour.
- The videoconferencing system could be used experimentally for a doctor–patient project; patient education was simulated here by a two-way-connection.
- Board meetings were organised by videoconferencing.
- In parallel to the videoconferencing, medical image files were transferred. In the projects discussed below, endoscopic images (Tele-ENT), images of the ocular fundus (early diagnosis of diseases), X-ray images (teleradiology) and pathological slides (telepathology) were transmitted.

In the following, the individual sub-projects are discussed with a focus on facilitators and barriers.

##### Tele-Tumour Conferencing

**Figure 5:**
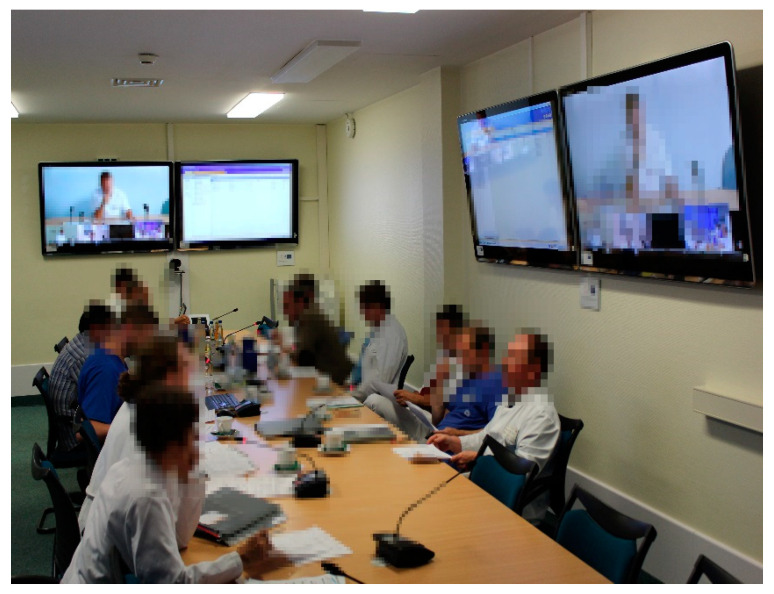
Tele-Tumour Conferencing. A sophisticated subproject included the tumour board of Eberswalde hospital, where smaller hospitals presented their cancer patients to specialists. Seated at the conference table are oncologists, a radiation oncologist and a radiologist. A pathologist in Pasewalk is discussing cases with a referring physician from the Templin hospital, approximately 65 km away. Documents, X-rays and pathology slides can be viewed by all participants simultaneously.

Every week, a tumour conference with a regional focus on Eberswalde took place, which connects several hospitals and several modalities (Figure 5). On average, 16.4 patient treatments were discussed in each session. The conferences were attended by an average of 7.9 doctors from various disciplines within the hospital, and 1.4 external specialists from other hospitals were consulted for consultations. The project was scientifically evaluated with business economists. As an example of an objective outcome parameter, the break-even-point for a regional tumour board was calculated at 272 patients discussed per year (main outcome indicator, details in [10]).

The videoconferencing tumour project has been operating without interruption and without external funding since 2012. Facilitators were economic benefits (travelling costs saved) and convenience (travel time saved by doctors). Videoconferencing allows for one physician to be “present“ at multiple sites nearly simultaneously. Interoperability was not fully achieved during the installation of the videoconferencing-network: the acquisition of only one “bridge” unnecessarily restricted the initiation of videoconferences to the Eberswalde site and made it difficult to further expand the technology. The acquisition of two additional bridges, costing a middle five-digit amount, would have been easily possible from the generous funding. This was missed by the site planning this subproject (Eberswalde) and resulted in the domination of the network by Eberswalde. 

##### Tumour Videoconferencing within a Hospital

The videoconferencing network on the German side was built around three larger hospitals. Greifswald (videoconferencing units in five clinics and institutes), Neubrandenburg (videoconferencing units in two clinics) and Eberswalde (videoconferencing units in seven affiliated hospitals/locations). Tumour conferences within one hospital are characterised by varying degrees of contribution to the discussion from the participating disciplines. While oncologists and radiotherapists usually provide information on all patients discussed, this is not the case for the diagnostic specialties. Pathologists often have only brief verbal contributions, with short demonstrations of sections. Tumour conferences at Greifswald only call the pathologists’ video stream into the conference when they are actually required to make a contribution. This scenario also applied to Eberswalde. 

A facilitating factor here is the time saved by the pathologist. This advantage was so obvious that the project was immediately accepted by all involved. One barrier was the need to link different videoconferencing-systems (in-house and inter-hospital). The causes for this were differences in the design of hospitals’ in-house meeting-room systems by the hospital administrations and also the inter-hospital purchasing of videoconferencing systems connecting different hospitals from project funds. 

##### Patient Education for Informed Consent via Videoconferencing

In populated regions, patients have to come to a treatment site twice: once to give informed consent and then again for the implementation of the procedure. The reason for this is the often legally required period of consideration of 24 h that must be granted to patients. The acceptance and effectiveness of getting patients’ informed consent via videoconferencing was investigated in a prospective study [11]. 

A facilitating factor was saving patients from a second trip to the hospital by creating the possibility of giving informed consent via a virtual meeting. The benefits—for example, saving sick or vacation days—were so important for patients that they agreed to see their doctor only on-screen. Unexpectedly, a second facilitating factor was the improved concentration of patients on screen, which led to a better memory of the educational contents. Barriers to widespread use were the lack of a uniform technological solution (hospital and at home for patients), difficulties for older patients who were inexperienced with the technology and doubts regarding the security of the video connection. 

##### Board Meetings on Videoconference

**Figure 6:**
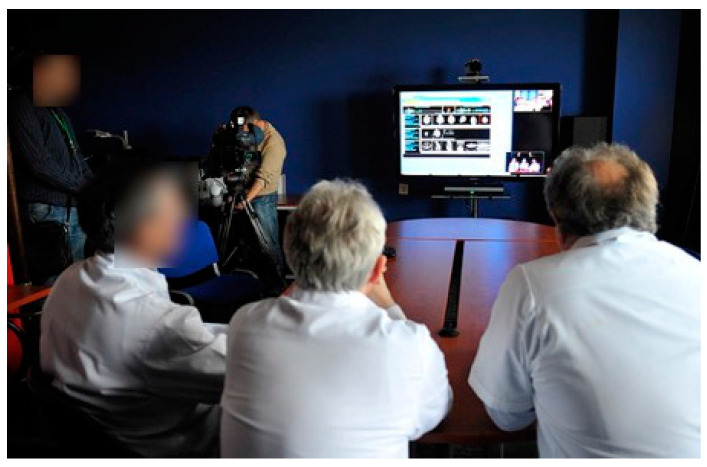
Cross-border meeting (Szczecin/Pasewalk). A working group of the project (in the front of the picture, three participants) and the German members (with modality pictures). From [12].

The sponsored area has a north–south diameter of about 250 km. In the east–west direction, the diameter is marginally larger, at 280 km. The meetings of the various boards (Figure 6), which took place monthly for years, previously required considerable travel activity. Even short conferences resulted in a significant loss of working days due to travel times (about 20 days per year). In the course of the project, the meetings were converted to a presence-to-videoconferencing-ratio of 1:3. Facilitators and barriers were the same as in the other applications: As might be expected, this changeover was easy due to the installed videoconferencing-network; acceptance was high among all parties involved. However, the acquisition of only one “bridge” unnecessarily restricted the initiation of videoconferences. 

#### 3.2.2. Teleradiology

**Figure 7:**
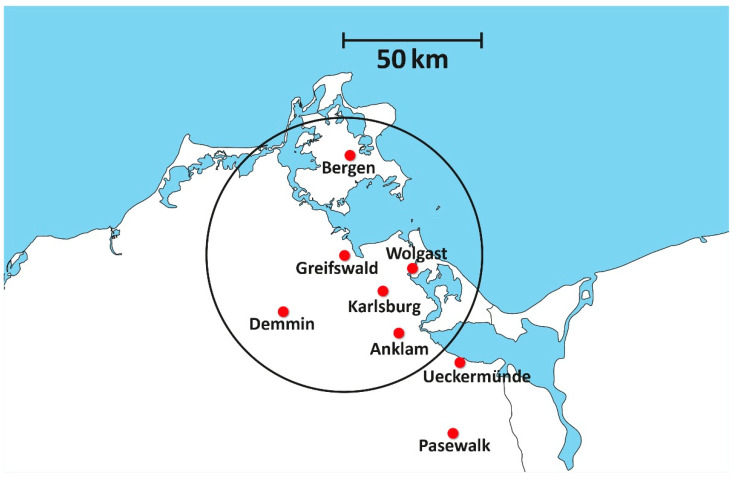
Geographical distribution of the German teleradiology network. The network has been running for more than 15 years. In Greifswald, there is a 24/7 radiological service in the university hospital. The surrounding city names represent the locations of connected houses that are also supplied in the network. According to German law, the backup method for downtime in teleradiology is a radiologist going to the relevant hospital and performing the examination there. Due to this restriction in German law, teleradiology was limited to hospitals that could be reached within an hour (Demmin and Karlsburg were not financed by the project). Conclusion: The loss of catchment areas of the hospitals due to a new territorial delimitation can be increased by the telemedical expansion of catchment areas. Telemedicine thus leads to better access to doctors in territorial states and to the better utilisation of medical services in the same regions (black circle, unbroken: catchment area of Greifswald University Hospital’s pathology department with telepathology. Red arrows: pathology connections.).

In sparsely populated regions, patient care is usually provided by a hospital structure with houses of different sizes. Even in smaller houses, the larger disciplines such as internal medicine and general surgery are often staffed with enough doctors to make 24/7 coverage possible. This does not apply to medical specialties such as radiology, and is even less applicable to pathology. In the studied region, only the hospitals in Greifswald and Neubrandenburg could offer radiological services 24/7. There are two difficulties: if there is a 24/7 radiology service in a sparsely populated region, it is often underutilised; however, in order to operate a German hospital with an emergency department or an intensive care unit, computed tomography diagnostics (CT) must be available 24/7. Teleradiology can compensate for these two situations. This increases the area in which on-call radiologists can provide their medical expertise in the night and on weekends. They are therefore utilised more efficiently by the teleradiological services and the hospitals that use teleradiological services can provide 24/7 CT diagnostics for emergencies. On the German side, the aim in the field of teleradiology was to establish a teleradiological 24/7 network, the development of contracts between supplier and customer and the economic evaluation of the costs that must be reimbursed in order to enable an economic operation, as well as a quality evaluation of the services. A cooperation between the University Radiology Greifswald and seven hospitals was established (Figure 7). On the German side, equipment had been installed in previous phases of the project. On the Polish side, radiological departments were newly equipped with scanners that allowed the digitisation of X-ray images. Several radiology departments in the sponsored houses were given radiological digital workstations consisting of high-resolution monitors and computers. Medical equipment (intraoperative MRI and X-ray workstations) was financed at two hospitals. Computer networks were added to all houses to enable the creation of a digital workflow.

In summary, teleradiology was permanently translated into a sustainable network, and its actual costs were scientifically assessed (main outcome indicator, details in [13,14]). The following figures may give an idea of the scope of services offered permanently; currently, there are approximately 1000 radiological exams per year reported during night and weekend shifts by one institution. A cost analysis of teleradiology from a provider’s perspective was performed using Monte Carlo analysis. Costs of reporting head and abdominal CT were calculated in a cooperation with academic economists (with €61.35 as the minimal charge for a head CT report to avoid losses). An increase in the catchment area for radiologists result in the better use of this profession’s services during night and weekend shifts.

The facilitating factors for teleradiology were the indispensability of the 24/7 service; the interdisciplinary setup of the project team; the presence of a chair in economics with a focus on medicine, who took over the economic evaluation, as well as the presence of an in-house counsel with a special focus on the legal aspects of teleradiology; and the digitisation of the participating clinics in previous phases with the establishment of a network. Another facilitator was the close cooperation between radiologists all over the area, since a large proportion of the radiologists working in peripheral institutions had been trained in the bigger centres. 

There was a chance missed by not supporting the system with weekly videoconferencing between the radiologists in the centres and the surgeons and internists in the area’s peripheral regions. This probably will be a barrier to further expanding the service.

#### 3.2.3. Telepathology

**Figure 8:**
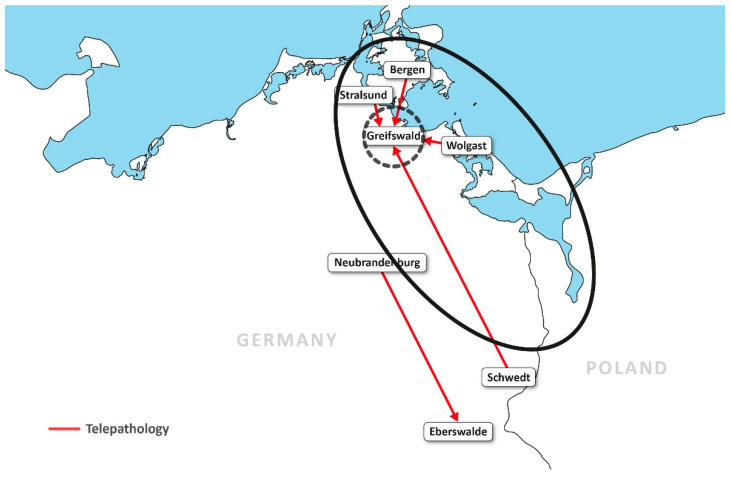
Enlargement of the catchment area of medical facilities through telemedicine. The inner, broken circle shows the direct catchment area of the pathology department at Greifswald University Hospital. Outside working hours, it is limited to the immediate area. Telepathologically, the catchment area is basically unlimited from a purely technical standpoint. The red arrows show telepathology connections in the pathology network.

In sparsely populated regions, most hospitals do not have their own pathologist. The conditions here are even clearer than in radiology. Of the three centres on the German side of our project, only two have a pathology department; none of the smaller houses do. The pathologists provide surgical departments with rapidly processed slides during operations and the reprocessing of surgical material after the operation. The slides are time-critical, as patients remain in anaesthesia until the results are communicated to the surgeon. Telepathological projects create slides cooperatively, using sophisticated technology during surgery (for example, pathologists directing the surgeons regarding from which part of the resectate specimens should be taken). Referring centres can digitally transmit scanned slides and have them evaluated in a pathology department. In our project, providing pathological services for all houses that required them through telepathology was the goal (outcome indicator). Two different approaches (Figure 8) were chosen for this purpose: firstly, Neubrandenburg Hospital’s pathology institute permanently provided a pathologist in a branch office in Eberswalde Hospital. Since this pathologist could not work at full capacity due to low case numbers, he was additionally providing telepathology services for the mother institution in Neubrandenburg; secondly, pathologists in Greifswald evaluated rapidly processed slides via telepathology for the hospitals in Bergen, Wolgast, Schwedt and Stralsund (the establishment of the service was the main outcome indicator; for details of the analysis, see [15]). 

Equipment on the German side was financed by the project. On the Polish side, the main focus was on funding pathological (and radiological) equipment. In Szczecin, Gryfice and Poznań, scanners for digitising pathological sections were procured and connected for remote consultations.

The Greifswald project has been scientifically evaluated. Retrospectively, the diagnostic accuracy of intraoperative frozen section telepathology was evaluated. It was highly acceptable at 98.95%. The average time for the preparation of virtual slides ranged from 10.58 ± 8.19 min. Investment costs were lower than those of robotic microscopy [15].

**Figure 9:**
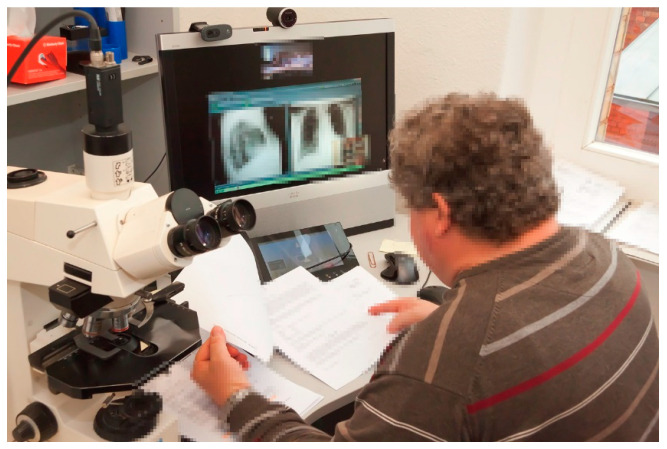
Telepathology. The telepathology workstation of this pathologist shows the pathologist’s desk during a tumour conference. He can work on his microscope while following the conference on his monitor. During the few minutes that the pathologist is needed in a typical teleconference, he may be seen and heard and show slides (see Figure 5). The higher productivity that is achieved in this way is particularly important given the few pathologists commonly available.

A facilitating factor was the fact that a functioning system was already available from the beginning of the project phase. This is not to be taken for granted, because pathology has a very high volume of data; i.e., it requires connections with high bandwidth and a high storage capacity. This barrier is caused by the high number of very thin cuts required for pathological evaluation and the bigger file size (compared to radiology) of the coloured sections (Figure 9).

#### 3.2.4. Tele-Ear-Nose-Throat (ENT)

**Figure 10:**
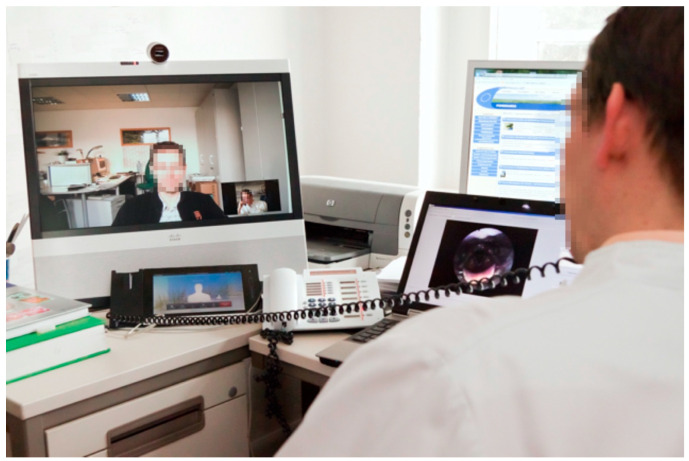
Video endoscopy during an ENT consultation. A non-specialised physician inserted the endoscope and images were automatically transferred to the specialist in a university hospital—in our project, between Templin and Greifswald. The distance between the two cities was roughly 150 km, and the driving time would be nearly 2 h. Specialised diagnosis was thus possible despite the distance, and therapy recommendations could be given. The Greifswald ENT specialist in the image is seen from the back, the general physician in Templin is shown on the monitor of the videoconferencing unit, and the image generated by the endoscope is shown on a smaller monitor to the right.

In the sparsely populated state of Western Pomerania, the economic efficiency before the funding period was poor for the few existing ENT departments. The Tele-ENT subproject consisted of videoconferencing and video endoscopy (Figure 10) between the patient and on-site doctor at the presentation site of the patient in the periphery and the ENT doctor in the centre (Greifswald). Videoconferencing was used to improve communication. The actual tele-ENT diagnostics were carried out with the help of a tele-endoscope. According to the plan [16], the on-site service doctor was able to insert the endoscope into the patient’s throat or into the outer ear canal. The endoscopic image was transmitted over the network to the university’s ENT department, where the doctor on duty verbally directed the endoscope and used the transmitted images for diagnostics. Specialist medical expertise could therefore be provided to any external location with a primary doctor and appropriate equipment on-site. The outcome indicator was the number of patients assisted with tele-endoscopy for ENT disease, assuming improved quality as a result (the establishment of the service was the main outcome indicator; for details, see [16]). 

A facilitating factor was the high quality of the endoscopy devices and the videoconferencing images. Barriers were experienced in two ways: the physicians working on-site used endoscopies—for example, for gastric examinations—and were accustomed to introducing the endoscope through the patient’s open mouth, while ENT physicians usually insert the endoscope through the patient’s nose. This difference, which seems trivial to the outsider, could not be surmounted in practice. Medical staff feared malpractice claims; the retraining of medical staff in peripheral sites should have been considered. This barrier has been reported in other studies as well [17]. Contractual solutions with external funding should have been worked towards. Another especially crucial barrier was the fact that the treating doctors in the periphery reported feeling devalued by the specialist support. The procedure was evaluated through interviews. A commonly mentioned argument was “We can care for our patients on our own!”. 

The difficulties could possibly have been eliminated with help from the medical centre, given sufficient will. The technology appears to be useful, especially in even more sparsely populated locations than our region. 

#### 3.2.5. Teleophthalmology Screening

Teleophthalmology screening made apparent another difficulty of telemedical projects. The project planned to use an existing Optical Coherence Tomograph (OTC [18,19])/telefundoscopy system. The system visualises the different retinal layers including blood vessels and the optic nerve head. Images are transferred in automated form to a center, where an experienced clinician can then evaluate the study. Screening for diseases such as increased arterial blood pressure or glaucoma is thus possible. The system was installed for Greifswald University Hospital and a hospital 50 km away without an ophthalmology clinic. The peripheral clinic thus provided its patients with a screening service. The outcome indicator was the statistical recording of a sufficient number of early diagnoses. The reduced number of necessary treatments and follow-up costs should be offset against the costs of maintaining the service. The outcome was not reached.

A facilitating factor was the pre-existing, technically evaluated system that was successfully installed. There was a barrier, however, which could not be overcome: the lack of funding of the prevention project—for example, as a pilot by health insurance companies. The necessary cooperation between the various professional groups (administration, the medical profession and health insurance providers) was not a given, and no contractual arrangement was reached. This should have been required and the possibility to purchase the equipment should have been used as an incentive.

#### 3.2.6. Tele-Stroke Diagnostics

Tele-stroke diagnostics consist of the transmission of clinical findings or a neurological examination of a patient. For the neurological examination, an assistant must be available on-site with the patient. The neurologist communicates and directs necessary examinations and observes the result via the videoconferencing device. 

Tele-stroke is a good application of telemedicine. However, it was less suitable in the project presented here, which was based on (and took its influence from) the financing of cost-intensive infrastructure/technology. The cost of equipment for tele-stroke is limited. However, tele-stroke projects must achieve a very well-functioning division of labour between doctors on duty (internists/surgeons), neuroradiologists, neurologists and interventional neuroradiologists. Independent from the project presented here, a Berlin-based tele-stroke project was established and permanently financed in a German innovation project [20]. Greifswald as a location is part of that independent project; the Pomerania telemedicine project only provided equipment in one of the hospitals. 

A facilitating factor of this and similar projects is the high treatment pressure: patients who are diagnosed and treated early often have a very good outcome, while not being treated within hours may result in death. The barrier for Pomerania was that, while the initial investment needs are relatively low, telestroke projects have high requirements concerning organisation and long-term-financing. However, Pomerania’s interest in tele-stroke possibly motivated neurologists to agree on a project realised by their own profession instead of a radiology-based project. This competition between specialists turned out to be a very potent facilitator.

### 3.3. Results Regarding Management Issues 

#### 3.3.1. Special aspects of a Cross-Border, Binational Project

At the start of the project in 2001, different specialties and regions were unevenly developed with regard to medical services. The project originated between two pathologists located in Pasewalk, Germany (10,000 inhabitants) and Poznań/Poland (536,000 inhabitants); the latter is not even located in an area in which the EU usually funds Interreg projects. Digitisation is a prerequisite of telemedicine in order to provide medical services over a distance. That each side of the project was allowed to start from their own point of development, rather than implementing identical infrastructure in both countries, was an important facilitating factor. Great efforts were made to secure data privacy at this stage. However, the solutions developed later turned out to be unfeasible. Nevertheless, in retrospect, it was crucial to simply start with what was possible.

Facilitating factors were a personal relationship between the founders of the project, the small scope of the initial project and the right timing, with digitisation only beginning in pathology and radiology when the project began. Board meetings by physicians and administrators from different German and Polish hospitals were considered the most rewarding aspect of the project, and this was a facilitator in its own right. 

An important barrier at a later stage was binational communication. While German was a common language between the founders of the project, this was not the case for all participants. Law offices, retired diplomats, translators and other organisations exist, which give professional help in cooperation between different countries. However, the leaders of the project, with its public funding, were reluctant to assign the very high fees that specialised law offices commanded. In retrospect, this was incorrect, and a solution should and could have been found by negotiation. For a time, a law office that specialised in German and Polish law provided this service by pointing out basic mistakes which are all but incomprehensible in retrospect; the lack of a Polish translation for the German association’s statutes was one such mistake. This probably made it impossible to co-opt Polish members into the association. A translator was present at board meetings, but this was no substitute for a more comprehensive service.

An academic position in psychology, anthropology, etc. financed by the project could have been an important addition to ease integration. Two full-time positions (one in-house counsel, one geographer) were financed for five years by EU project funding, and one, for three more years by the participating hospitals (in-house counsel). 

#### 3.3.2. Participation of Multiple Hospitals

An “association” is easily established in Germany, with no capital necessary. It may be tax-exempt, as was the case here. It had serious drawbacks, as associates were not always aware of the financial risks. This led to the telemedicine project being perceived as “not-for-profit” or “pro bono”, at least by the participating physicians, while in effect it was a company with considerable financial risk and statutory liability (ranging into an eight-digit sum). It was obviously vital for the project to responsibly handle financial matters and to communicate this to the public. All investments had to be pre-financed by the participating hospitals, with the association later receiving 90% of the funds. The hospitals paid a percentage of the overhead according to the percentage of the EU funding they received. One problem with the accounting was that all of the reimbursements were via the German side and in Euros. Therefore, as this then had to be converted into Polish Złoty, this was a considerable financial risk.

In summary, the formation of an association with a large number of participating hospitals was itself deemed a barrier. While the association is preserved as a mantle under a new board for possible use in the future, the project described here was developed differently. An attending clinician at Greifswald University Hospital (Holger Lode, paediatrician, specialised in neuroblastoma treatment) with a highly specialised area of work and existing referrals from Poland was chosen. His approach received Interreg funding.

#### 3.3.3. Project’s Legal Issues

The experiences in telemedicine obtained in the project were partially transferred into the national law of both countries. In the beginning of the project, the legal situation of telemedicine as an innovative medical discipline was—with a few exceptions—unregulated in both Germany and Poland and therefore unclear for the acting hospitals, hospital administrators and physicians. The undefined legal situation was a barrier for all project actors. Legal expertise in the project was a facilitating and essential factor. In connection with the project, telemedical questions that arose were legally processed [21]. During the project´s duration, first regulations for telemedicine were created in Germany and Poland. This circumstance shows that the EU is able to influence, through its projects, national framework and even national health systems, for which the EU has no real legitimisation. Furthermore, the undisputed phrase that law follows the reality of life was confirmed.

Another legal aspect of the project was transporting pilot projects into routine care. Physicians tend to cooperate based on personal trust, and this may help with starting pilot projects. To integrate telemedicine into everyday use, contracts have to be drawn between hospitals (teleradiology, telepathology) or between healthcare providers and hospitals. This last aspect was neglected in the ophthalmology and otorhinolaryngology projects, and these two projects faltered after funding ran out [16,19,20]. In the same way, it does not make sense to give public funding to modalities that are ultimately privately owned. Mammography screening is a multi-million Euro program owned by private practices in Germany, and an attempt to create a comprehensive storage structure for the program was futile [22].

Calling for bids was organised by a specialised law office. Law students were employed to prepare and handle the calls. This worked very well. Procedures were established in this manner, as well as the documentation of bids and contracts awarded.

Associations according to German law were registered. The structures providing this service correspond to parts of local courts. As they were alien to Eastern Germany when the project was first conceived (similar structures did not exist in the German Democratic Republic), they did not function well and were a permanent nuisance to the project. The influence of the project, however, was large enough to achieve improvements with support from local politicians.

Problems arising during the implementation of the project were voiced at binational government meetings by the project’s chairman.

## 4. Discussion

A review of telemedical literature in NIH PubMed does not reveal many multinational, cross-border medical projects. The reasons for this may be the close connection between medical care and a common language between doctor and patient (large price differences between medical services in the border area for lifestyle interventions such as dental care, cosmetic surgery and hair transplants are certainly an exception). The EU considers the goal of cross-border medical care as a building block for the creation of a data network. To this end, it supports projects from neighbouring regional states—in the presented example, Germany and Poland. The prerequisite is the existence of a large city in the development area—in the example, Szczecin, as the historical centre of the region.

Some working groups have named facilitators and barriers for the implementation of telemedicine projects. However, these reports often concern doctor-to-patient telemedicine. A more general recommendation is found in a manual [3] that identified facilitators for the introduction of telemedicine:

1. Existence of a master plan at state level that is well coordinated and financially resourced.
2. Infrastructure data security.
3. The presence of an electronic patient record with interoperability.
4. Adapted legislation.
5. Reimbursement.

Standardised procedures, on the other hand, were not considered necessary.

Brady et al. [4] described in 2021 how publicly available data can be used to prioritize ophthalmic telemedicine. Their work can be understood as the identification of a facilitator. Zanaboni et al. [5] described the early status of Norwegian telemedicine projects and above all identified sparsely populated states as facilitators for the use of telemedicine. This observation can be applied to our project. The same authors [6] later described facilitators for the routine use of telemedicine in Norwegian hospitals. They identified an economy of scale with greater benefits derived from very large telemedicine projects. To this end, the authors reviewed different networks with figures for numbers of per capita consultations. A lack of resources and political guidelines, especially those relating to reimbursement, were described as barriers. A paper from Hawaii [7] proposed three recommendations for improving medical care in unevenly populated areas that suffer from a lack of doctors. As a facilitator, the establishment of a business model to reduce complexity is suggested. A second point concerns the retention of doctors. The approach of the authors is in line with our experience of coaching and training doctors who have remained in the region through telemedical access to specialists on neighbouring islands. This was the rationale of our Tele-ENT project. 

The underlying principle of telemedicine is, in short, to expand the catchment areas of medical services. In the Interreg phase described here, which lasted until 2020, this was partially achieved separately on both sides of the border. In Germany, this mainly concerned radiology and pathology as well as tumour conferences, while on the Polish side, pathology and radiology structures were established. 

An expansion of the catchment areas of medical offers in the international area makes sense in the case of highly specialised therapy for rare diseases. Accordingly, a paediatric-led, cross-border project for the care of children with neuroblastoma was designed and financed for the next project phase. The EU goal of cross-border care might thus be achieved in a highly specialised and very small, but nevertheless important, field of medicine. A legal framework for cross-border medical services remains desirable. 

## 5. Conclusions

The following recommendations can be given for doctor-to-doctor telemedicine projects with high investments in telemedical infrastructure:

- The establishment of telemedical infrastructure must often be asynchronous in large areas, but always in cross-border projects. The causes are the different stages of development at the beginning of the project.
- Before investing, the financing of future ongoing operations should be secured. An interdisciplinary setup of the project team in EU funded projects is essential.
- Market power in the purchase of expensive technology is an important argument for large infrastructure projects.
- Publicly funded infrastructure projects often require a financial commitment from beneficiaries in the project; in the case shown here, this was 15%. This is ineffective, as 15% of projects that have already been planned by applicants can always be added to applications. Thus, no additional funds in fact have to be raised for the funded projects. It would make more sense to demand from beneficiaries that they add 15% to 25% of the total costs for manpower, supporting the transition into daily practice.
- Cross-border telemedicine projects should have professional counselling from academic institutions or specialised law offices. A law office may also prepare binding contracts, which should be signed before the rolling-out of equipment.
- Projects involving competing hospitals tend to suffer from being labelled as “altruistic”, which is not a strategically beneficial term in societies founded on economic success. Input into government decision-making and into regional government authority was a way to resolve this “flaw”.

## Figures and Tables

**Table 1:** Subprojects. Note, different goals for the German and Polish sides. Outcomes and outcome indicators are given and facilitators and barriers are listed. See details in text.

Subproject	Goal Germany	Goal Poland	Main Outcome Indicator	Outcome	Facilitators	Barriers	Soft Facilitators/Barriers
Tele-tumor conferencing.	To establish a twice-weekly online-only video conference with multiple hospitals and multiple specialties.	-	Economic analysis of working conference.	Established successfully, in permanent full use, economically sound.	Only way to establish tumor conferences in areas with low population density.	-	Implemented by chairman of large hospital in project, prestige project.
Patient’s informed consent.	Scientific evaluation.	-	Feasibility.	Feasible.	Obvious advantage of avoiding time and expenses for travel.	Interoperability problems.	Patients remembered content better.
Tele-conference for board meetings.	To avoid travelling to board meetings.	To avoid travelling to board meetings.	-	Established successfully, used when necessary.	Obvious advantage of avoiding time and expenses for monthly travel to board meetings.	Binational meetings too sterile, bonding an important factor for the success of the whole project.	-
Tele-radiology.	To establish 24/7 computed tomography (CT) reporting coverage in German area. Scientific evaluation	To provide digital X-ray equipment.	Establishment of service, equipment delivered, assessment of cost-effectiveness.	Teleradiology established successfully in Germany. Digital X-ray equipment provided to hospitals in Poland. In use.	Teleradiology in off-hours without alternative: no emergency department without computed tomography access (!).	Legal restriction in Germany at time of implementation.	Radiologists in area known to each other from training.
Tele-pathology.	To establish 24/7 pathology coverage in the German area.	To provide digital pathology equipment; tele pathology service.	Establishment of service, equipment delivered.	Telepathology established successfully in Germany and Poland. Also used for teaching.	High cost of digitisation of pathology; funding from project was a very strong incentive.	Low acceptance of telepathology in one provider. Abandoned by one providing hospital as management did not want to support competitors.	Little alternative for providing hospitals, as pathology departments not economically feasible for smaller hospitals.
Tele-earnose throat (ENT).	To establish 24/7 ENT specialty coverage in the German area.	-	Establishment of service, equipment delivered.	Project was technically implemented, later discontinued.	Technology available, was installed successfully; not enough patients for a university department in this area of low-population density.	Doctors at receiving hospitals not familiar with placement of endoscopic device via nose: legal problems expected.	Smaller hospitals not willing to accept specialty support, prefered to provide for their patients without outside help.
Teleophthalmology.	To establish early diagnoses from retina scans by screening in one hospital.	-	Establishment of service, equipment delivered.	Establishment of tele-screening in hospital, evaluated in university clinic, later discontinued.	Technology was available, was installed successfully.	Program established technically, but no access to financial re-imbursement. Screening.	Started by personally acquainted department heads, stopped when one of them left.
Tele-stroke.	To establish a tele-stroke network.	-	-	-	Pomerania perceived as competitor by neurology. Very strong incentive for neurology to implement own project.	Low-cost of technology employed. No funding necessary. Aspects of organizing services prevailed.	Clinical specialty joined Berlin project, established successful program with minimal funding by Pomerania.

## Data Availability

Not applicable.
